# Hysteroscopy in the Evaluation of Intrauterine Pathologies With Primary and Secondary Infertility: A Prospective Study From North India

**DOI:** 10.7759/cureus.101116

**Published:** 2026-01-08

**Authors:** Lubna Inam, Mohammad Shazib Faridi

**Affiliations:** 1 Obstetrics and Gynaecology, Hamdard Institute of Medical Sciences & Research, New Delhi, IND; 2 Urology, Dr Baba Saheb Ambedkar Medical College and Hospital, Delhi, IND

**Keywords:** diagnosis, female infertility, hysterosalpingography, hysteroscopy, infertility

## Abstract

Introduction

Infertility remains a significant obstacle in reproductive health, affecting countless couples worldwide. Hysteroscopy is essential in the evaluation of infertile females. Therefore, this study has been conducted to evaluate the role of hysteroscopy in the evaluation of intrauterine pathologies in Indian women with primary and secondary infertility.

Methods

This was a prospective observational study conducted at a tertiary care hospital for 18 months. Ninety infertile females aged 18-40 years with normal husband semen parameters underwent infertility workups and were divided into primary and secondary infertility. All patients underwent both hysterosalpingography (HSG) and hysteroscopy. The findings were recorded, and the suspicious lesions were sent for histopathological examination.

Results

There was a statistically significant difference in mean age, mean duration of infertility, and BMI between primary and secondary infertile females. HSG showed abnormalities in 12 (19.7%) patients in primary and 8 (27.6%) patients in secondary infertility. However, 21 (34.4%) females in the primary and 10 (34.5%) in the secondary infertility group had pathologies on hysteroscopy. HSG was 54.8% sensitive and 94.9% specific with a positive predictive value (PPV) and negative predictive value (NPV) of 85% and 80%, respectively. The accuracy of HSG was 81.1%. The endometrial anomalies were the most common finding, 21 (23.2%), on hysteroscopy, and endometritis was the most common, 6 (6.6%), on histopathology (HPE). With respect to HPE, hysteroscopy had 100% sensitivity, 66.7% specificity, with 81.8% PPV, NPV as 100%, and an accuracy of 88.2% in this study.

Conclusion

Hysteroscopy is a useful procedure in diagnosing uterine pathologies that often remain undetected with USG and HSG. However, hysteroscopy alone cannot rule out tubal blockage. Hence, in female infertility, a complementary use of HSG and hysteroscopy is recommended.

## Introduction

Eight to twelve percent of couples around the world have difficulty conceiving a child at some point in their lives, and 1 in 6 couples have infertility in their lifetime [1,2]. Female infertility is on the rise today [3], and the prevalence is increasing owing to changes in lifestyle and social norms of marriage and conception. 

Infertility is defined as the involuntary failure to achieve pregnancy after at least one year of regular unprotected intercourse or therapeutic donor insemination, and even after six months for women over 35 years of age [4]. Infertility is termed primary if conception has never occurred and secondary if there is failure to conceive after having achieved a previous conception, including ectopic pregnancy [5]. In India, the prevalence of infertility is 8% of all married couples [6].

The basic investigations for female infertility include assessment of cervical, uterine, tubal, and ovulatory factors. Uterine abnormalities, congenital or acquired, are implicated as one of the important causes of infertility. Uterine factors are accountable for up to 16.7% of patients [7-9].

The patients undergoing in vitro fertilization have shown that in the presence of uterine cavity anomalies, the pregnancy rates are lower [10], and correction of these anomalies has been associated with improved pregnancy rates [11]. Therefore, endometrial cavity assessment has been included in the evaluation of infertile couples, and uterine lesions may cause infertility by interfering with proper embryo implantation and growth [12].

The goal of uterine cavity evaluation is either to obtain a sample of the endometrium or to diagnose structural abnormalities [13,14]. At present, the hysteroscopy is considered the gold standard in evaluating the intrauterine pathologies, which could be as high as 16% in infertile females [15]. Hence, this study has been conducted to evaluate the role of hysteroscopy in the evaluation of intrauterine pathologies in Indian women with primary and secondary infertility.

## Materials and methods

This was a prospective observational hospital-based study conducted in the department of Obstetrics & Gynecology at a tertiary care center over a period of 18 months after obtaining approval from the Institutional Ethics Committee (ELMC/EC/R-Cell/2013/247). The inclusion criteria were patients aged 18 to 40 years with primary or secondary infertility with a normal menstrual cycle and normal semen parameters of the husband. The exclusion criteria were active pelvic infection, contraindication to hysteroscopy, severe cardiopulmonary compromise, and those who opted out of the study.

In total, 101 patients were recruited in the study, of whom 90 were included in the final analysis. Eight patients were lost to follow-up, and in three patients, the surgeon was not able to negotiate the hysteroscope in the uterine cavity. A detailed history, physical examination, and laboratory investigations such as complete blood count, urine analysis, urine culture and sensitivity, thyroid profile, prolactin level, abdominal ultrasound, hysterosalpingography, and hysteroscopy findings were recorded in the proforma specially designed for the study. The abnormal findings were endometrial or uterine pathologies, whereas hysteroscopy with no uterine pathology was considered normal.

Technique

After obtaining written informed consent, hysteroscopy was performed in the early proliferative phase (day 7 to day 10) of the menstrual cycle under spinal anesthesia. The patient’s bladder was emptied, and they were placed in the lithotomy position.

Under all aseptic precautions, Sim's speculum was inserted into the vagina, and vulsellum was used to hold the upper cervical lip. The internal os was dilated, if required. The hysteroscope is then introduced through the external os and guided through the endocervical canal into the uterine cavity under visual control. The uterine cavity was systematically inspected for tubal ostia, the right and left cornua, fundus, anterior, posterior, and lateral walls. Whenever endometrial pathology was noted or suspected, a biopsy was taken, and tissue was sent for histopathology and TB PCR. Photographs were systematically obtained, and details were recorded of all the patients undergoing hysteroscopy.

Device specification

All procedures were performed using a Diagnostic Double-Flow hysteroscope, consisting of a 4 mm and 30-degree STRYKER telescope and a 4 mm double flow sheath with an integrated irrigation channel. Illumination was provided by a high-intensity cold light source via a fiberoptic lead. Normal saline was used as the distending medium. Uterine distention was accomplished by a pressure infusion cuff. The pressure was pre-set to 80-100 mmHg.

Statistical analysis 

The data was analyzed using IBM Corp. Released 2009. IBM SPSS Statistics for Windows, Version 15. Armonk, NY: IBM Corp., and subsequently, data was entered into Microsoft Excel (Redmond, USA). The outcome was compared using the chi-square test. The confidence level of the study was kept at 95%; hence, a "p" value less than 0.05 indicated a statistically significant difference.

## Results

**Figure 1:**
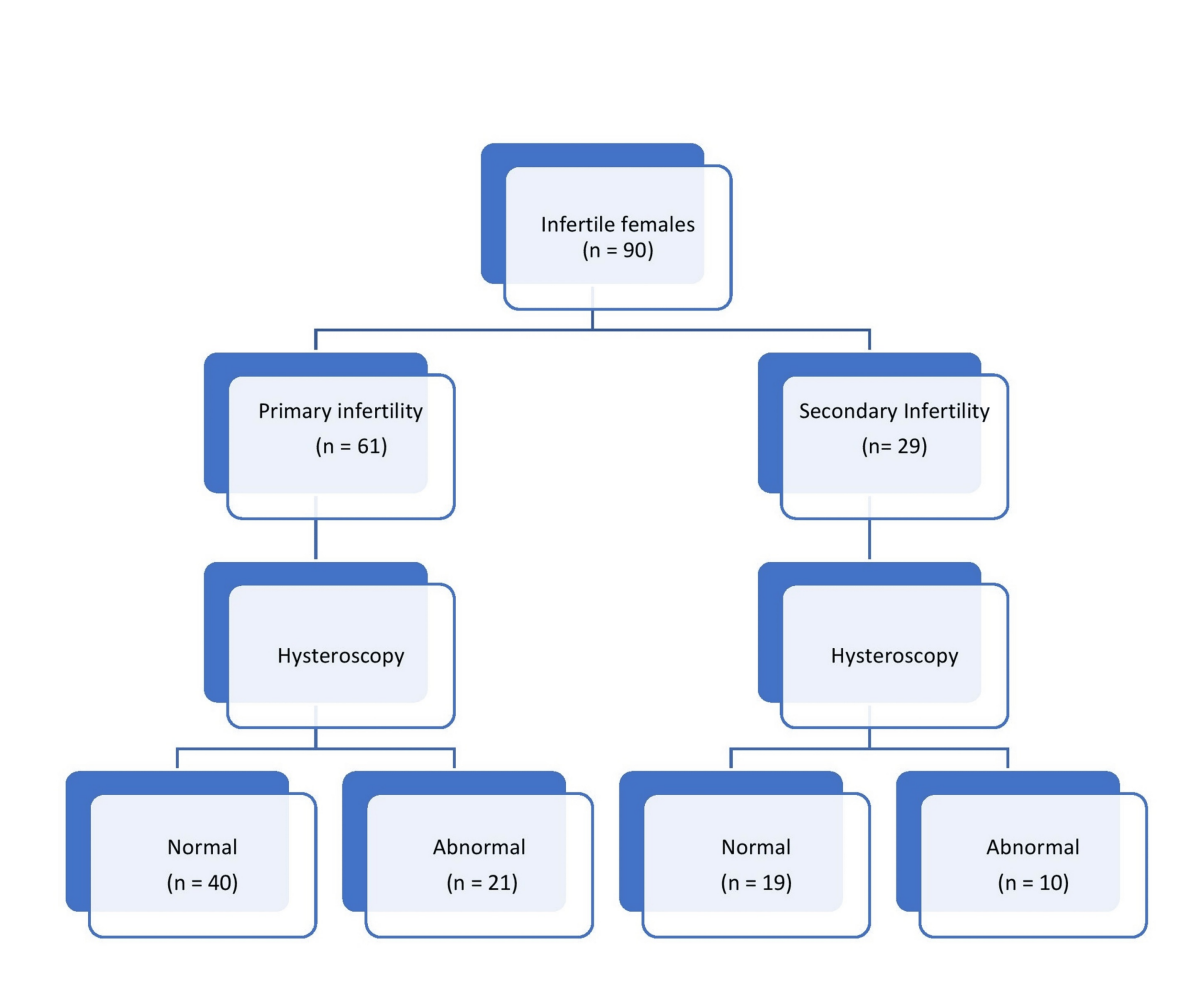
Flowchart of studied females

A total of 90 infertile females falling in the sampling frame of the study were enrolled; 61 (67.8%) were cases of primary infertility, while the remaining 29 (32.2%) belonged to secondary infertility (Figure 1). There was a statistically significant difference in mean age (29.75 ± 2.06 vs. 31.48 ± 2.90, p=0.002) and mean duration of infertility (4.24 ± 2.43 vs. 6.41 ± 2.93, p < 0.001) between primary and secondary infertile patients. Moreover, we also observed a significant difference in the BMI of the primary and secondary infertile women (24.92 ± 2.70 vs. 26.26 ± 2.93, p = 0.036) (Table 1). In our study, the majority of the patients belonged to the upper middle-class group (as per the Kuppuswamy socioeconomic status scale) in both primary and secondary infertility females, 51 (83.6%) vs. 22 (75.9%); however, the difference was not significant (p = 0.190).

**Table 1:** Demography of the patients BMI: Body mass index

Parameter	Total (n=90)	Primary Infertility (n=61)	Secondary Infertility (n=29)	p-value
Mean Age ± SD (years)	30.27 ± 2.46	29.75 ± 2.06	31.48 ± 2.90	0.002
Mean duration of marriage ± SD (years)	4.94 ± 2.78	4.24 ± 2.43	6.41 ± 2.93	<0.001
Mean BMI ± SD (Kg/m^2^)	25.35 ± 2.83	24.92 ± 2.70	26.26 ± 2.93	0.036

On evaluation of infertile patients on hysterosalpingography (HSG), 20 (22.2%) patients had abnormal findings, with 12 (19.7%) patients in the primary infertility group and 8 (27.6%) patients in the secondary infertility group having pathologies (Table 2). The difference was not statistically significant between primary and secondary infertility groups (p=0.399) on HSG findings.

**Table 2:** Abnormal findings on hysterosalpingography

Outcome	Total (n=20) N (%)	Primary infertility (n=12) N (%)	Secondary infertility (n=8) N (%)
Arcuate uterus	1 (1.1)	1 (1.6)	0 (0.0)
Bilateral cornual blockage	4 (4.4)	2 (3.3)	2 (6.9)
Bilateral tubal blockage	6 (6.7)	4 (6.6)	2 (6.9)
Bicornuate uterus	3 (3.3)	2 (3.3)	1 (3.4)
Septate Uterus	1 (1.1)	1 (1.6)	0 (0.0)
Unilateral tubal blockage	5 (5.6)	2 (3.3)	3 (10.3)

In the studied population, 21 (34.4%) patients had abnormal findings in the primary infertile women, and 10 (34.5%) had abnormalities in the secondary infertile females on hysteroscopy (Figure 1), but the difference was statistically insignificant (p = 0.996). The endometrial anomalies were the most common finding: 21 (23.3%) on hysteroscopy, irrespective of the type of infertility, and endometritis was the most common in 6 (6.6%) among endometrial abnormalities on histopathology (HPE) (Table 3). With respect to HPE, hysteroscopy had 100% sensitivity, 66.7% specificity, a positive predictive value of 81.8%, a negative predictive value of 100%, and an accuracy of 88.2% in this study.

**Table 3:** Abnormal findings on hysteroscopy

Outcome	Total Patients (n = 31) N (%)	Primary Infertility (n = 21) N (%)	Secondary Infertility (n = 10) N (%)
Endometrial abnormalities			
Endometrial hyperplasia	2 (2.2)	2 (3.3)	0 (0.0)
Endometrial polyp	3 (3.3)	3 (4.9)	0 (0.0)
Endometritis	6 (6.6)	5 (8.2)	1 (3.4)
Fibrosis	4 (4.4)	1 (1.6)	3 (10.3)
Intrauterine synechiae	5 (5.6)	2 (3.3)	3 (10.3)
Intrauterine synechiae with fibrosis in the cavity	1 (1.1)	1 (1.6)	0 (0.0)
Congenital uterine abnormalities			
Arcuate uterus	1 (1.1)	1 (1.6)	0 (0.0)
Subseptate uterus	2 (2.2)	2 (3.3)	0 (0.0)
Uterine didelphus	1 (1.1)	0 (0.0)	1 (3.4)
Uterine septum	1 (1.1)	1 (1.6)	0 (0.0)
Cervical stenosis	5 (5.6)	3 (4.9)	2 (6.9)

In our study, in comparison to hysteroscopy, HSG was only 54.8% sensitive and 94.9% specific, with a positive and negative predictive value of 85% and 80%, respectively. The accuracy of HSG against hysteroscopy was 81.1%.

The most common complication post-hysteroscopy was pain, 6 (6.7%), followed by mild bleeding per vaginum, 5 (5.5%).

## Discussion

For decades, HSG has been regarded as one of the most common initial investigations in the evaluation of female infertility. It helps in the assessment of fallopian tubes and the uterine cavity, but is associated with significant false reports [16]. Given the failure to achieve an accurate picture of the uterine pathology in cases of infertility, a minimally invasive technique, hysteroscopy, has gained popularity in a short span of time, which is a safe, simple, and effective technique in the evaluation of the uterine cavity and diagnosis of associated abnormalities in infertility workup [17].

In the present study, among the infertile women being evaluated, primary infertility (61, 67.8%) was more common than secondary infertility (29, 32.2%). Similar results were reported by a large meta-analysis, with 51.5% of patients diagnosed with primary infertility [18].

In the present study, the mean age of women with primary infertility (29.75±2.06 years) was significantly lower than that of women with secondary infertility (31.48±2.90 years). Similarly, the mean duration of marriage was significantly lower in the primary infertility group (4.24±2.43 years) as compared to the secondary infertility group (6.41±2.93 years). Our findings were in agreement with another study where females with secondary infertility were older and had been married for longer [19]. Likewise, the mean BMI of women in the secondary infertility group (26.26±2.93 kg/m²) was significantly higher than that of the primary infertility group (24.92±2.70 kg/m²). A higher BMI of women in the secondary infertility group could be attributed to the relatively higher age or fat deposition during the first pregnancy. These findings corresponded with the previous literature [20].

Tubal and cornual blockage are major reasons for female infertility, and HSG is a useful radiographic modality in diagnosing tubal and cornual blockage, which is otherwise missed on hysteroscopy. In our study, a total of 20 patients were diagnosed with pathologies on HSG-12 (19.7%) in the primary infertility group and 8 (27.6%) in the secondary infertility group. Tubal and cornual blockade was seen in 15 (16.7%) cases, and uterine abnormalities in the remaining five cases. All five cases of uterine diseases were confirmed on hysteroscopy. Our study showed that eight (13%) patients had tubal blockage in the primary infertility group, whereas seven (24.1%) in the secondary infertility group had it on HSG. Our results are similar to published data by Al Subhi T et al., where 22 (19%) women with primary infertility and 30 (29%) with secondary infertility had tubal blockage [21].

In contrast, hysteroscopy revealed 31 (34.4%) cases with cervical, congenital uterine, and endometrial abnormalities, in which endometrial pathologies were most common (21, 23.2%). Among endometrial diseases, endometritis (6, 6.6%) was the most common. Parveen et al. attributed endometrial diseases to the cause of infertility in 11 (17.6%) of their series comprising 62 patients [22]. In this study, a total of 21 (34.4%) of primary and 10 (34.5%) of secondary infertility patients had uterine abnormalities, but there was no significant association between the type of infertility and hysteroscopic findings. Hysteroscopy provided additional information about uterine and endometrial abnormalities in 12 (13.3%) cases in whom tubal/cornual blockage was already reported by HSG. The study published by Sahu L et al. showed similar results that hysteroscopy had no significant difference in detecting the uterine pathologies in women with primary and secondary infertility, 77 (33%) and 36 (39%). A recent study also confirms that HSG is an effective tool in assessing tubal patency, while hysteroscopy provides better results in diagnosing uterine abnormalities [24].

In this study, the histopathology (HPE) was done in 17 (18.9%) cases (12 primary, 5 secondary). The abnormalities were revealed in 9 (75%) primary infertility patients, only 2 (16.7%) cases of each chronic endometritis, endometrial hyperplasia, and endometrial polyps, respectively, and 3 (25%) cases of tuberculous endometritis. With respect to HPE, hysteroscopy had 100% sensitivity, 66.7% specificity, a positive predictive value of 81.8%, a negative predictive value of 100%, and an accuracy of 88.2%. These findings confirm that hysteroscopy has an excellent diagnostic efficacy in evaluating the endometrial cavity and provides information that is not obtained by blind endometrial sampling.

Our study also revealed HSG was only 54.8% sensitive and 94.9% specific; it had a positive and negative predictive value of 85% and 80%, respectively. The accuracy of HSG against hysteroscopy was 81.1%. Siam et al. reported that the accuracy level of HSG is similar to that of the present study, 81.6% against hysteroscopy. They also highlighted that significant uterine pathology remains undiagnosed in a sizeable proportion of infertile women undergoing HSG evaluation as compared to hysteroscopy [25].

Limitations

We had some limitations also. It is a single-center design study, which may explain the relatively small size of the study group. Moreover, in our study, patients had to be fit from an anesthesia perspective, as all hysteroscopies were done under regional anesthesia. Furthermore, it is a descriptive study, so no comparison was done between different techniques.

## Conclusions

Thus, the present study showed that hysteroscopy is a useful procedure in the diagnosis of endometrial, uterine, and cervical pathologies that often remain undiagnosed with conventional imaging tools like USG and HSG. However, at the same time, the use of hysteroscopy alone cannot rule out the diagnosis of tubal and cornual blockage. Considering the important role of both these factors in female infertility, a complementary use of HSG and hysteroscopy is recommended.
